# A Novel Telemetry System for Real Time, Ship Main Propulsion Power Measurement

**DOI:** 10.3390/s19214771

**Published:** 2019-11-02

**Authors:** Michał Bonisławski, Marcin HOŁUB, Tadeusz Borkowski, Przemysław Kowalak

**Affiliations:** 1 Electrical Engineering Department, West Pomeranian University of Technology, Szczecin, al. Piastów 17, 70-310 Szczecin, Poland; michal.bonislawski@zut.edu.pl; 2 Maritime University of Szczecin, Mechanical Engineering Department, Wały Chrobrego, 1-2 Szczecin, Poland; t.borkowski@am.szczecin.pl (T.B.); p.kowalak@am.szczecin.pl (P.K.)

**Keywords:** shaft power measurement, telemetry systems, shipbuilding industry, wireless power transmission

## Abstract

Modern ships are required to increase the energy efficiency and minimize fuel consumption. This paper presents the construction, main properties and exemplary measurement results of a novel system intended for main shaft power monitoring. The telemetry system consists of the stationary part, responsible for wireless supply energy transfer to the rotating part. Additional functions of the stationery unit include radio-based, bidirectional communication with the rotating, microcontroller-based unit, and Modbus-based communication with the graphical user interface. The non-stationary (rotating) part receives the necessary energy using the wireless transmission and performs the torque and speed measurement using strain gauge and a special setup of the wireless energy system. A novel system of flexible printed circuit board (PCB) coils is used for wireless energy transmission and increases the flexibility of the device while minimizing the necessary size, weight, and costs of the setup. The microcontroller unit used for measurements allows proper sampling of highly dynamic signals and can be used for advanced drive system diagnostics or as a typical power monitoring device. Such unit was installed on a ferry and operation was monitored for several sea trips. Main results depict characteristic power data referenced to vessel speed and specific fuel oil consumption (SFOC). Proposed system construction allows to reduce system costs and provides stable readings for long period of operation.

## 1. Introduction

Ships energy efficiency, associated to air pollution and greenhouse gas emissions, has been an issue considered within International Maritime Organization (IMO) for a considerable time. The international Convention for the Prevention of Pollution from Ships (MARPOL) Annex VI was adopted in 1997 [1], at that time mainly focusing on air pollution, in particular NO_X_ and SO_X_ emissions.

The next step of environmental action was greenhouse gas emissions reduction. In 2011, IMO adopted resolution MEPC.203(62) [2], next suite [3,4,5,6] of technical and operational measures which together provide an energy efficiency framework for ships. These mandatory measures entered into force on 1st January 2013, as Chapter 4 of MARPOL, Annex VI. Further amendments to those requirements mean that ship types responsible for approximately 85% of carbon dioxide (CO_2_) emissions from international shipping are to be subject to strengthening requirements for energy efficiency and, together, they represent the first-ever, mandatory global regime for CO_2_ emission reduction in maritime sector. The 2015 Paris Agreement under the United Nations Framework Convention on Climate Change (UNFCCC) sets the goal to contain the rise in average global temperatures to well below 2 °C above pre-industrial levels and to pursue efforts to limit it to 1.5 °C. It is a historic and legally binding global climate agreement adopted by 195 countries. 

Considering the developments at international level, directive of the European Union (EU) 2018/410 [7] mandated the EU to review the progress achieved in the IMO towards an ambitious emission reduction objective, and on accompanying measures to ensure that the sector duly contributes to the efforts needed to achieve the objectives agreed under the Paris Agreement. The Directive called for action to address shipping emissions from the IMO or the EU to start from 2023, including preparatory work and stakeholder consultation. Following the Paris Agreement, in April 2018 the IMO adopted the initial strategy on reduction of greenhouse gases (GHG) emission from ships, setting several carbon intensities as well as total GHG emissions reduction targets, coupled with a vision for the full decarbonization of the sector, and a list of possible short-, mid-, and long-term further measures to achieve such objectives. 

The EU Regulation on the monitoring, reporting, and verification of carbon dioxide emissions from maritime transport (Regulation 2015/757 [8]), established a robust system for the monitoring and reporting of verified data on annual fuel consumption, CO_2_ emissions, and other energy efficiency-related parameters for ships, calling at European Economic Area ports, from 1st of January 2018 onwards. Its primary objective is to promote the reduction of CO_2_ emissions in a cost-effective manner. Similarly, to the EU Monitoring, Reporting, and Verification system (MRV) of CO_2_ emissions, the IMO adopted a mandatory data collection mechanism which entered into force on 1st of March 2018.

The existing IMO instruments dealing with ship energy efficiency include both technical and operational measures defined in Annex VI of the MARPOL Convention. Although these measures set a mandatory limit on the Energy Efficiency Design Index (EEDI) for new ships of 400 GT and above and mandate the use of the Ship Energy Efficiency Management Plan (SEEMP) for all ships of 400 GT and above, it is clear that significant and timely strengthening of these instruments is needed to make the required impact on the reduction of GHG emissions from international shipping. 

As a consequence, development of the ship and its propulsions is determined by current trends in maritime transport, especially in the scope of energy efficiency and emissions. Significantly established emission reduction targets, as defined by IMO and EU, now set new directions for ship design and operation. Significant attention is paid to ships energy efficiency, as it stimulates the relevant emission reduction of all exhaust components; CO_2_, NO_X_, SO_X_, PM, and HC. Historically, shipping is permanently engaged in efforts to optimize fuel consumption and ships are known as the most fuel-efficient means of cargo transportation. However different studies identified a significant potential for further improvements in ships energy efficiency mainly through the use of new technologies such as: Electronically controlled engines, combined propulsion systems, and alternative fuels e.g., liquefied natural gas, hull design, or waste recovery systems. Although the easiest way to improve ship energy efficiency is speed reduction, but there is a practical and safety limit, not to mention cargo logistic issues. Ship fuel oil consumption represents a significant part of the total operating cost. Ship performance still can be optimized, and operational energy efficiency measures should be employed either at sea, while steaming, or in port during berthing. 

The SEEMP is mandatory for all ships including non-transport, such as service ships or dredgers. The conventional transport ships, such as container carriers, tankers, and bulk carriers, are easy to categorize as they are intended for a single purpose, with a single design point specifying a given cargo load and design speed. The main goal of SEEMP on board each ship is to give specific guidance to ship staff to improve ship energy efficiency and to reduce its total fuel consumption if applicable. Proper technical management can provide energy conservation opportunities by means of main propulsion upkeep in peak technical condition and working mode optimization from energy efficiency point of view. Effective implementation of SEEMP requires constant or periodical measuring, monitoring, documenting, and reporting. 

Usually main propulsion performance evaluation during normal operation is done as part of the SEEMP. The purpose is to assess the long-time performance of the system as well as to assure that the equipment works as expected. Typically, existing performance monitoring tools are used, either manually or in automated setups. Energy management as a tool for increasing ship energy efficiency must contain measurement of energy consumption and monitoring of performance indicators. Exemplary measuring instruments following SEEMP technical task that can be used include:

- main propulsion power meters (including shaft torque and rotational speed meter),
- bunkered and consumed fuel flowmeters, preferably mass flowmeters,
- ship monitoring system of electric energy distribution,
- voyage monitoring system of the ship (speed, draught, trim, and wind).

Monitoring of SEEMP implementation and its efficiency should be supervised and analyzed by company dedicated office. For best-practice in energy management, energy efficiency, and ships performance goals need to be defined based on the energy efficiency measures (EEM) being implemented. These goals are subsequently used for benchmarking purposes and evaluation of the efficiency of the measures employed. Wherever possible, goals are quantitative and time-based. Review and evaluation of achievements is carried out on a planned regular basis. Efficient tools of self-evaluation and improvement are target (goals) setting and subsequent evaluation (e.g. after one year) of EEM implementation.

This paper gives an overview of a modern, novel main propulsion power meter. A compact design is proposed implementing state of the art wireless power transmission, radio based data transfer, and a user friendly, graphical user interface (GUI). Proposed solution is smaller than systems available on the market and does not require main propulsion shaft disassembly. Specific design aspects of the prototype are presented and exemplary measurement results are shown.

Paper is structured in a following way: Overall system construction is presented in Section 2 with detailed discussion on measurement principle (Section 2.1), the construction and main properties of the stationary part (Section 2.2), the design and construction of the wireless power transfer system (Section 2.3). Section 2.4 gives an overview on the non-stationary (rotating) part and Section 2.5 provides basic data related to the GUI and final system form. Exemplary measurement results are presented in Section 3. Section 4 summarizes main properties of the system and outlines main conclusions. 

## 2. Materials and Methods

**Figure 1:**
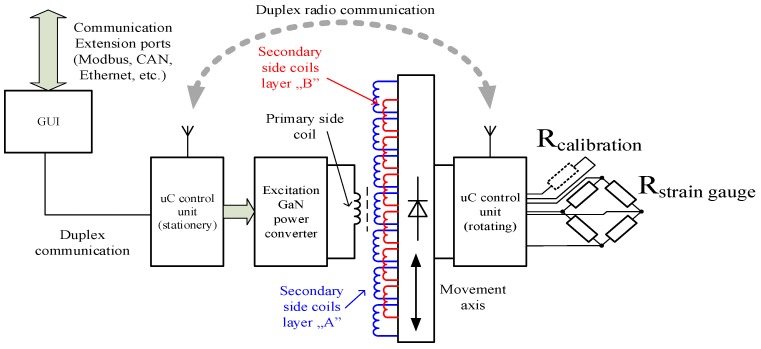
Overall system construction of a proposed, real time shaft power telemetry unit.

Proposed real time, main shaft power telemetry system consists of three main components: The stationary part (responsible for wireless supply power transfer, bidirectional radio-communication with the non-stationary part and Modbus communication with the graphical user interface), the rotating part (responsible for measurement conduction and system calibration, radio communication with the stationary part) and a touch-panel based GUI. Overall system construction is schematically depicted in Figure 1.

In order to discuss the properties of basic modules in more details following subsections will introduce most important aspects of operation of individual parts of the system.

### 2.1. The Torque Measurement Principle

The torque generated by the driving engine may be determined in two ways: By measuring the energy consumed by the driven device, or by determination of the transmission shaft strain or twist. The first method is widely applied in various test bench, where the energy is consumed by a dynamometer, but it is impracticable for monitoring of the ship propulsion engine load. The second method might be applied instead. In the applicability of the Hook’s law, the loading torque *T* is proportional to the drive shaft strain ε:

(1) $$T=\frac{\varepsilon \cdot \pi \cdot G\cdot D^{3}}{8}$$

The same torque is twisting the shaft by the angle *φ* on a measured shaft length *L*:

(2) $$T=\frac{\varphi \cdot G\cdot J_{0}}{L}$$

where:

*L*—shaft length [m],
*T*—torque [Nm],
*D*—shaft diameter [m],
*G*—shaft material shear modulus [Pa],
*φ*—twisting angle [rad],
*ε*—shaft strain [—],
*J*_0_—polar moment of inertia of the shaft section [m^4^].

Practically, the operation principle of all torque meters utilized in shipping is based on above two equations. In case of the Equation (1), the strain is usually measured by a thin foil strain gauge bonded to the shaft surface [9,10]. That technology is used by a number of commercial torque meters providers [11,12,13]. More advanced methods of strain measurement, like laser-based interferometry [14,15], optic cameras [16,17], fiber Bragg gratings [18], or surface acoustic waves [19,20] are under development, however they are not widely used in the shipping industry yet. Over the last 20 years, concern on the torque detection based on the Equation (2) is observed. Its main advantage is unnecessity of the power supply for a rotating part of the system. Most of proposed methods are based on the phase shift measurement of two rotating code wheels mounted on the shaft over a shaft length *L* [21,22,23]. Number of commercial products based on Equation (2) are available on the market today [24,25,26,27,28], their main disadvantages is requirement of enough free length of the shaft. This necessary length may vary from 0.5 m to over 6 m depending on sensor type and shaft diameter. That amount of space needed disqualifies this method from application on drives with short shaft lines. Methods based on strain gauge offer much better compactness.

The accuracy and calibration of the measuring system poses another difficulty. Typically, the accuracy of commercial torque meters given by manufacturers is 0.1 to 0.25% of the full scale. That accuracy may be valid for instrumentation only in consequence for the measurement of the strain or twisting angle, respectively. The accuracy of the torque determination should take under consideration properties of the shaft which becomes a crucial component of the torque measuring system. Only very few commercial system producers [11] inform that the shaft properties should be counted in. Those properties are numerically expressed in a value of the shear modulus G. For steel the value of G is usually between 74 to 85 GPa and it may vary on the steel chemical composition and its thermal or mechanical treatment during production (like forging, rolling, quenching, or tempering). Based on values given in [29] the average value of the shear modulus for steel is 80.5 GPa with a standard deviation of 4.4%.

Every marine propulsion shaft must be certified for the steel composition and strength properties [30]. Classification societies require only a limited number of parameters [31] and there is no shear modulus neither even Young’s modulus nor Poisson ratio included at all. Only in some specific cases, usually on shipowner’s request, additional tests of the shaft material are carried out during the manufacturing process. Practically the *G* value is taken from common steel properties tables, which means it is approximated with very limited accuracy.

The strain gauge has number of disadvantages related to the bonding process and measuring uncertainty [32]. Nevertheless, presently that is the only available method allowing proper calibration of almost the entire measurement chain. Typical application of any type of sensor requires calibration of zero shift and the span. In case of marine shaft torque measurement both are practically difficult to process. Since all presented methods measure the twist angle or the strain rather than the torque. Even if the driving engine is stopped the shaft line remains under a residual stress or twist so zero shift measurement is not possible. Moreover, this residual stress differs from stop to stop because of the variable conditions of the shaft line. Therefore, calibration of the zero signal should be repeated every time the propulsion is stopped. Calibration of span by application of reference torque is practically impossible as the nominal torques in marine propulsion systems are from thousands to millions of Nm. Unlike other measuring methods in the strain gauge method a shunt resistor may be applied to simulate pre-calculated value of the torque. It should be understood, that even this way of span calibration is imperfect because there is still no proven reference value of the torque applied, but at least the entire electric circuit of the measurement system may be verified for correct readings. For this reason, the strain gauge method was chosen in this project.

### 2.2. The Stationary Part

The stationary part is meant to fulfil following, main tasks:

- Enable clear and multi-domain information flow of the measurement results to the graphical user interface using the Modbus protocol,
- Enable real-time, duplex (two-directional), and wireless communication with the non-stationary part,
- Introduce clear status information (power supply level of the non-stationary part, power supply level of the unit, and communication status) of the system operation to the user,
- Based on the feedback from the non-stationary part, control the operation of the wireless energy transmission unit.

**Figure 2:**
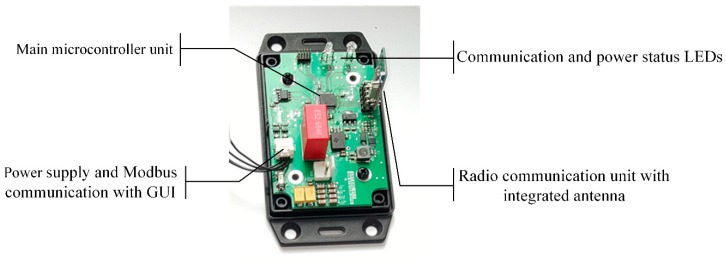
Construction of the stationary part with most important components marked.

In order to fulfill all these requirements a microcontroller platform had to be chosen with respective communication possibilities and sufficient computational power. The ST STM32L0 microcontroller platform was chosen for this application. Stationary part prototype construction is depicted in Figure 2.

The overall power consumption of the stationary part, including the wireless power transfer unit, is around 7 W. It is supplied from one phase marine grid using certified Siemens 6EP3331 series power supply.

### 2.3. The Wireless Power Transmission System

**Figure 3:**
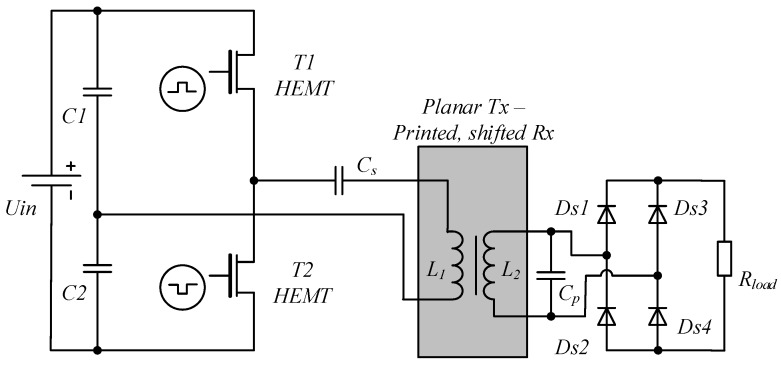
Topology of the wireless, series–parallel resonant power transfer system with GaN switches and planar coils.

In order to increase the flexibility of the system, minimize the size and allow highly-efficient operation series–parallel, resonant mode wireless supply power transfer topology was chosen [33] with frequency-mode control. Wide bandgap (GaN technology, high electron mobility transistor (HEMT)) switches were used to allow high frequency of operation as well as an increase of overall system performance [34]. Proposed topology of the wireless power transfer system is presented in Figure 3.

**Figure 4:**
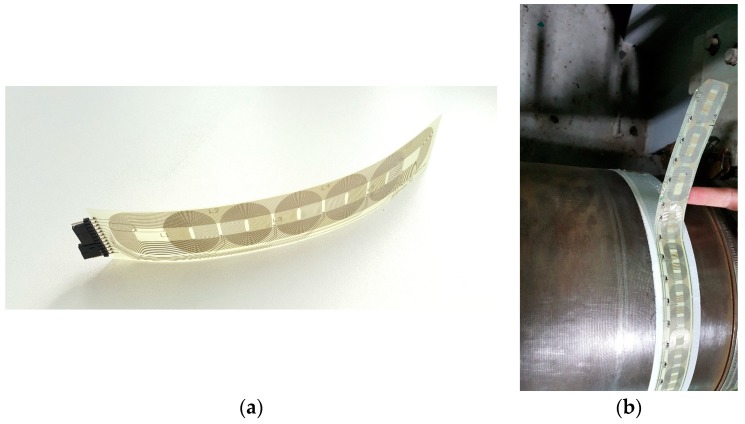
(**a**) Wireless power transfer secondary side (receiver *Rx*) coils and (**b**) their exemplary arrangement on a main, marine shaft.

Series resonance of the primary part allows semi–soft switching of the power switches while the parallel resonance of the secondary side increases the voltage amplitude of the non-stationary part. In order to propose a compact solution, coils of the wireless power transfer system are arranged on a flat planar core on the stationary side (the transmitter, *Tx*) while the secondary side coils (receiver coils, *Rx*) are prepared as a two-layer, flexible PCB printout [35]. *Rx* side coils and the coil arrangement on a shaft are presented in Figure 4a,b, respectively. One must carefully design the secondary side form as the electromagnetic field has to follow a path minimizing the parasitic, eddy current losses in the shaft material.

It should be noted that the coil arrangement has a phase shift between the upper and the lower layer to allow sufficient magnetic coupling for any primary–secondary side arrangement as well as successful power transfer when the shaft is in standstill. The *Rx* coils are prepared in a way that can be arranged as a series connection of an arbitrary number of single elements allowing system operation for shafts of varying diameters. More details on the wireless power transfer system can be found in [34]. An additional, important feature of the system is that one coil has independent terminals and a signal from this coil is used to calculate the rotational speed of the shaft with no additional sensors.

Main advantages of such coil construction include high energy transmission efficiency, redundancy (system can operate even if a number of single coils are damaged), operation while the shaft is rotating or in standstill, simple coil alignment procedure while mounting (status displays), low transmission system height after assembly, large gap between the stationery unit and the rotating coil (up to 15 mm), and pre-mount system preparation (minimized efforts on board).

### 2.4. The Non-Stationary Part

The non-stationary part is meant to fulfil following, main tasks:

- Perform the µV scale measurements on the strain gauge and rotational speed readings from the wireless power supply coils,
- Enable real-time, duplex (two-directional), and wireless communication with the stationary part,
- Introduce clear status information (communication status also indicating proper power supply levels) to the user,
- Based on the feedback from the stationary part, allow the calibration procedure based on a calibration resistor.

**Figure 5:**
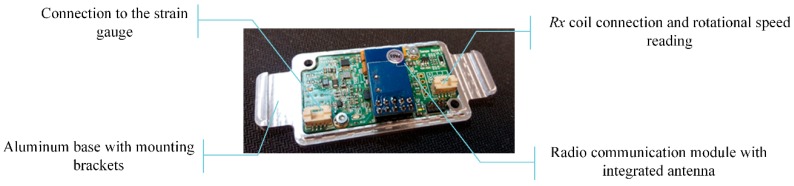
Construction of the non-stationary part with its main components.

Because the part is rotating the power consumption, size, and the weight of the unit are important design factors as well as proper mechanical enclosure allowing easy shaft mounting and clear status reading while assuring marine-standard mechanical ruggedness. Again, a low-power microcontroller unit is responsible for the operation of the unit (STM32L0). Its construction is presented in Figure 5.

Since the rotating part can be supplied in shaft standstill no additional power sources (batteries, supercapacitors) are necessary for continuous system operation. Such a device was already mounted on shafts of varying sizes with no mechanical difficulties.

Dynamics of the strain gauge reading is limited by the analog to digital converter used (ADS1220 from Texas Instruments, Dallas, Texas, United States) and can be set in a range between 20 to 2000 samples per second. From this perspective the analysis of shaft dynamic behavior (like mechanical shaft vibrations or dynamic torque production by the main motor) can be an interesting feature for some applications. The prototype presented measures the averaged shaft torque and the conversion resolution was set to 90 readings per second. The oversampling technique was used to increase the reading accuracy.

### 2.5. The Graphical User Interface (GUI)

**Figure 6:**
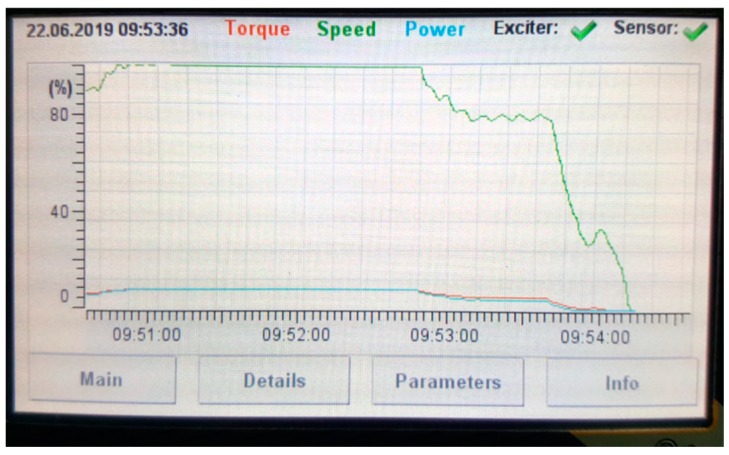
Exemplary reading of the graphical user interface (GUI).

The telemetry system has to present measured data in a transparent way while allowing measured signal post-processing (filtration, scaling, and time trend analysis). Because the system should offer the possibility to communicate with other marine control units from varying producers, communication extension ports can be provided offering analog (4–20 mA, 0–10V), or digital (Modbus, CAN, Ethernet, Seatals, etc) communication protocols. In addition, a SIM-card port can be provided for distant data reading and analysis. The GUI also provides real-time help while installing the system (for proper *Tx*–*Rx* alignment). An exemplary reading is presented in Figure 6.

To simplify the construction a touch-panel GUI with full programmable logic controller capabilities was selected. The chosen ABB–B&R (Bernecker and Reiner) system allows configurable communication with extension ports thus allowing system adoption for various applications. The system is supplied from the same source as the stationary part simplifying the overall complexity.

**Figure 7:**
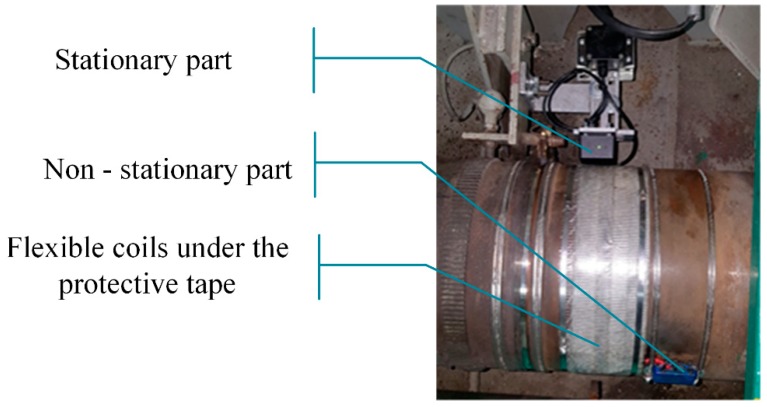
Prototype of the power measurement unit after the assembly on the main propulsion shaft.

A prototype of the system described in previous sections, after mounting on a main propulsion shaft, is presented in Figure 7.

## 3. Results and Discussion

### 3.1. Test Bed System Validation

In order to facilitate adequate validation of the torque measurement system test bed examination was made, using the test engine and water brake, with main specifications listed in Table 1. The test engine used was a four-stroke, medium-speed, turbocharged marine diesel engine.

**Figure 8:**
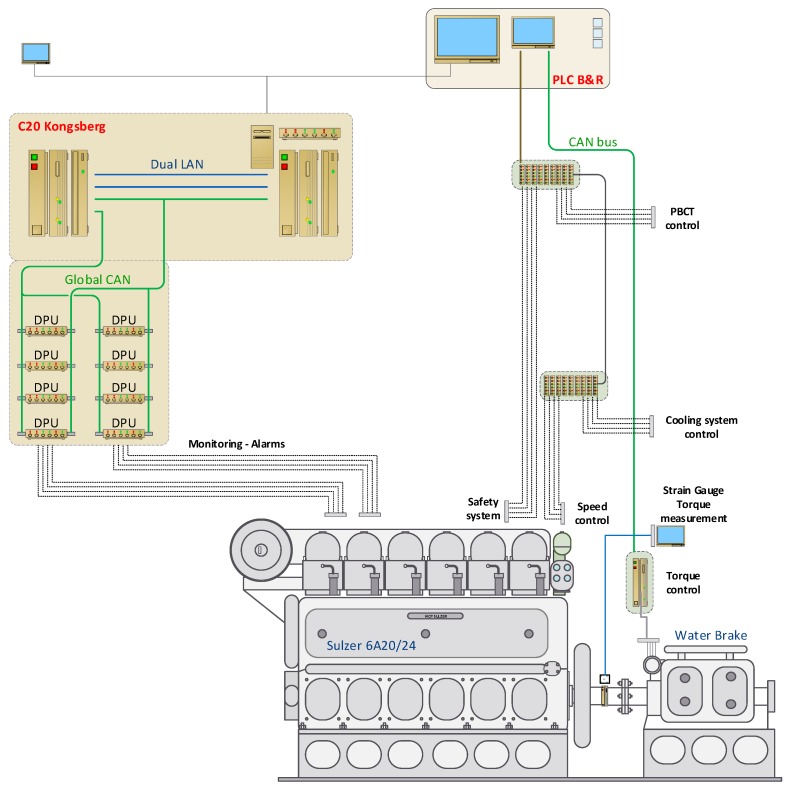
Engine test bed set-up configuration.

Comparison of delivered engine power was undertaken during the standard ISO–8178 test cycle E2 designed for marine main propulsions that includes generator drive over the speed-torque settings. In order to increase the number of standard four load points of the E2 cycle additional loads of idling and 90% when rising load and 85%, 65%, and 42% when load reducing, were tested. Torque indications at standstill conditions were compared too. The experimental set-up is shown in Figure 8. During the test cycle the engine was controlled and monitored by the means of its own, computer-based control and data acquisition system.

The water brake’s dynamometer is equipped with force transducer enabling torque calculation with aid of formula (x):

T = k∙*F_d_* (3)

where: *F_d_*—dynamometer measured force [Nm], k—dynamometer constant.

**Figure 9:**
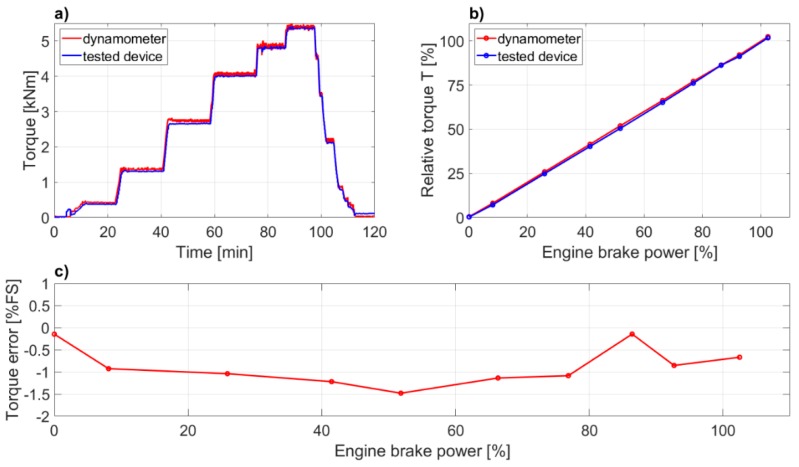
Comparison of proposed torque measurement device with the water brake dynamometer records: (**a**) time plots; (**b**) averaged values; and (**c**) proposed power meter device torque error in relation to dynamometer.

Consequently, the dynamometer torque measurement accuracy is equal to the force sensor accuracy. The comparison results of proposed power meter and water brake torque readings are presented in Figure 9a–c.

It should be emphasized, that the preliminary validation was meant to verify if the new device is capable to reproduce the torque signal with satisfactory accuracy comparing to the dynamometer. The difference in indication was assumed satisfactory for torque errors not bigger than *δ_T_* = ±3% of a full-scale range (FS), while the value of the nominal engine torque was adopted as the FS. As shown on Figure 9a,b, the new device measurement capability is not far from the dynamometer. The measured torque error (Figure 9c) was calculated from the formula:

(4) $$\delta_{T}=\frac{T_{s}-T_{d}}{T_{n}}\cdot 100\%$$

where: *δ_T_*—Torque error [% FS], *T_s_*—Torque measured by new device [Nm], *T_d_*—Torque measured by dynamometer [Nm], and *T_n_*—engine’s nominal torque [Nm].

The maximum error of —1.5% FS is observed at nearly 50% of engine load, which is better achievement than initially assumed. Additionally, a nonlinearity error was calculated with two methods: A maximum deviation from linearity with formula (5):

(5) $$u_{lin-max}=max\left|T_{s}-T_{fitted}\right|$$

and a standard error from linearity, with formula (6):

(6) $$u_{lin-std}=\sqrt{\frac{1}{n-2}\overset{n}{\underset{i=1}{\sum }}{\left(T_{s}-T_{fitted}\right)}^{2}}$$

where: *T_fitted_*—torque calculated with linear regression.

Results of 0.9% for maximum error and 0.6% of FS for standard error from linearity are very promising. The results obtained encourage to work further on the determination of the measurement uncertainty with a new device. Results of further investigations are planned to be presented in a separate article.

### 3.2. Ship Based Validation

To maintain the low operating costs of a merchant ship in a daily service is not only a duty required by SEEMP but also a challenge. Generally, the fuel consumption should be kept at minimum, which is possible if ship hull, main propulsion and machinery are in good condition. On the other hand, ships must be able to maintain their course in the open sea, maneuver safely in ports and restricted channels and to stop within a reasonable distance. These minimum capacities are required under any load condition, both at high speeds and at more moderate speeds which can be associated with restricted waters and in both calm and high sea conditions. Hence it is important to monitor ship performance; propulsion power, speed, and fuel consumption during the lifetime. The problem of conducting ship speed and powering trials have been addressed in several standards [36,37,38] and recommendations [39].

Typically, there is a well-defined ship and sea condition specified in contract between the owner and the shipyard and refers to ship steaming in calm weather. Later, the recorded data type for ship performance assessment are the same as those measured during the shipyard sea trials. However, there are important alterations: Actual hull resistance, non-optimal weather conditions, ship crew (not specialist), and the measurement instrumentation. It can be assumed that the prediction of full scale power of a ship is an essential part of ship performance assessment process.

**Figure 10:**
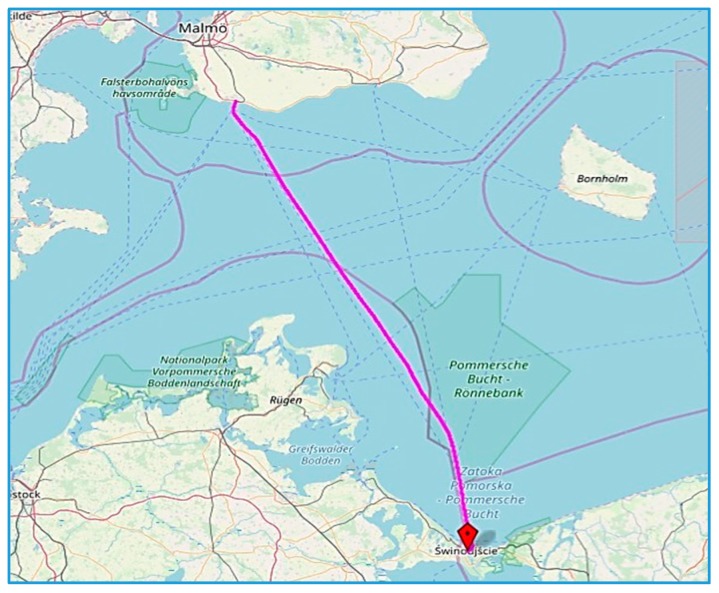
Ship single voyage trace.

As deliberated in Section 2 main propulsion power monitoring system was installed on a passenger Ro-Ro ship. Ship main specifics are given in Table 2. A Ro-Ro ship, that is driven by the means of two main propulsion units (specified in Table 3 and Table 4) is currently flying in short route within the Baltic Sea (Figure 10).

**Figure 11:**
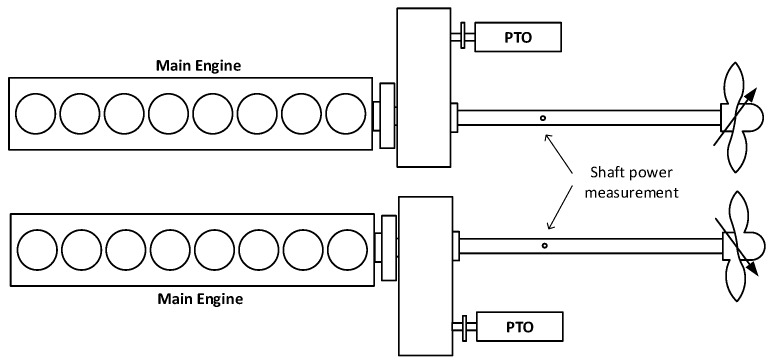
Ship main propulsion layout.

The ship is propelled by two medium-speed diesel engines and two controllable pitch propellers. The schematic propulsion layout is depicted in Figure 11. Also, two shaft power take out (PTO) generators are utilized during maneuvering and later a single one, while ship steaming. Main ship specifications are defined in tables below.

Proposed system prototypes record effective power on both propeller shafts and are additionally supported by ship operating parameter acquisition system which comprises real time signals including ship speeds over the ground and through the water, fuel oil consumption, ship trim and heel, meteorological conditions (wind speed and direction), and electric energy distribution.

**Figure 12:**
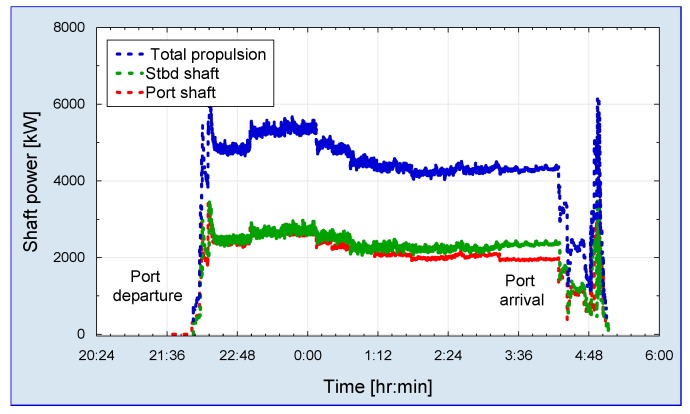
Single voyage propulsion power record.

Main propulsion data are kept in a storage device and the ship data are transferred with packet communication services. The data analysis enables measurement result verification with adequate voyage positional information obtained from global positioning system of the ship. Consequently, measured ship propulsion power creates base analysis for actual specific fuel consumption. Figure 12 shows time series plot of a single voyage as an example of recorded data.

**Figure 13:**
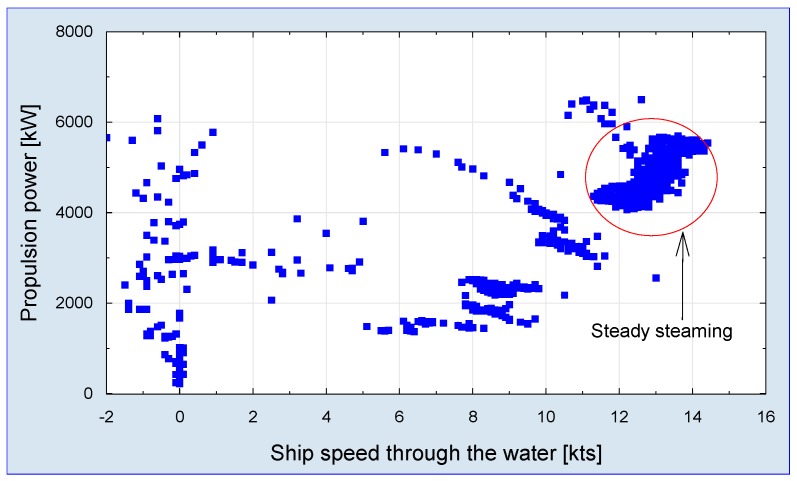
Ship propulsion load operational spectra during a single voyage.

For logistical reasons the vessel maintains a relatively low sailing speed, despite it’s designed main propulsion power. The ship is equipped with a log of speed measurement, then steady service performance of the ship can be estimated. The analysis covers the service period when wind direction and velocity affected ship speed and resulted in load fluctuations. Such exemplary analysis is presented in Figure 13.

**Figure 14:**
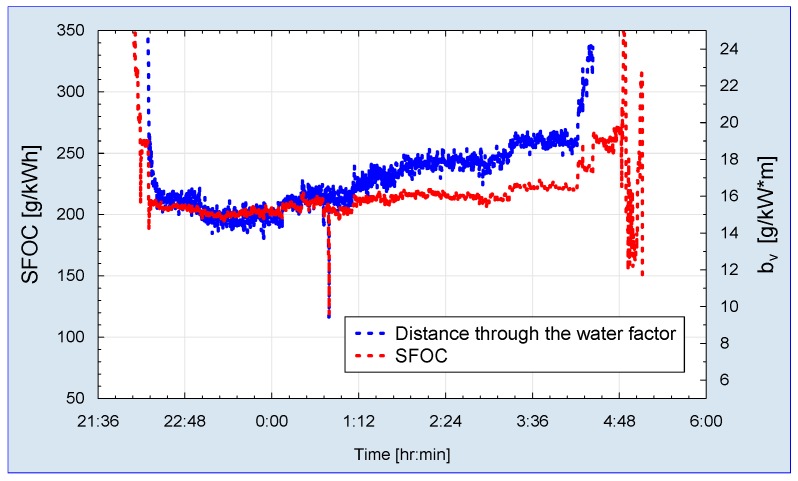
Time record of the specific fuel oil consumption (SFOC).

Essential part of the ship operation and economic valuation is defined by fuel oil consumption. Even sophisticated and expensive fuel mass flow measurements do not provide an easy base for energy efficiency analysis. The rate of fuel oil consumption was calculated based on the fuel oil consumption and main propulsion output power measurement. As shown in Figure 14 specific fuel oil consumption changes in relation to the ship speed and main propulsion output, respectively. In order to determine changes in the ship energy efficiency more adequately an indicator was proposed that reflects the changes of specific fuel oil consumption (SFOC) in relation to the ship distance traveled.

It is not proper to use the absolute ship distance traveled (over the ground) but it is necessary to use the ship distance with respect to water. The course of the indicator formulated in this way, shown in Figure 14, indicates the areas of optimal low SFOC, despite maintaining a fairly constant speed of the ship. Such a characteristic symptom is caused by the fact of the selection of engines for the main propulsion of the ship and the optimization of the efficiency of these engines for service sailing speeds of 18.5 kts. It is worth noting that this is a 27-year-old ship and engines from the ship construction period were typically configured this way.

## 4. Conclusions

Knowledge of the actual ship main propulsion load is currently the basic component of an energy-efficient operation method in the context of current and planned IMO regulations. The propeller shaft load is transferred accurately into fuel consumption and determines exhaust emissions. Next to accurate quantification powering characteristics and ship speed also are sensitive to a set of conditions such as ship and propeller condition, ship displacement, shallow water effects, sea state, and wind velocity. Consequently, these factors must be monitored and documented to the greatest extent possible.

Taking into account such circumstances a measuring system was designed and built for precise monitoring of the ship main propulsion power. The measuring system is based on the known and proven technique for assessing the torsional load of the shaft using strain gauges. The innovative solution is the contactless method of sensor electric supply and communication with data acquisition and control unit.

The monitoring system is characterized by very low electrical power consumption (7W) while being resistant to harsh operating conditions and maintaining excellent properties expressed by measurement accuracy. The power measurement system is calibrated taking into account *G*-modulus as specified by the shaft maker and preliminary zero offset is determined just prior to the trial. The shaft diameter used in the power calculation is derived from the shaft circumference in-situ and measured at the location of the torque instrumentation.

In order to summarize most important advantages of the system presented in this paper in comparison to commercially available torsional cells following properties can be underlined:

1. small system size, weight, and power consumption,
2. small, minimal shaft clearance for mounting (15 cm),
3. redundant wireless power transmission system with high efficiency,
4. regular measurement chain calibration possibility,
5. no shaft disassembly required for mounting,
6. configurable communication link to higher level monitoring systems,
7. user friendly GUI with data storage and post-processing functions,
8. low system cost.

Service performance of the novel measurement system was tested for several months by installing a system on a Ropax Ferry. As observed from the results, data obtained remained precise and stable for a long period. The data was recorded when the ship passed through the same sea area in order to examine the accuracy and bias error. The measurement system exhibits overall bias error smaller than 2%. The measurement unit proposed in this paper can be offered as standalone version or may be interlinked to other ship management systems.

## Figures and Tables

**Table 1:** Test bed engine and water brake specification.

**Engine**	
Type	Sulzer 6A20/24, in-line, non-reverse
Number of cylinder	6
Bore/Stroke [mm]	200/240
Rated engine speed [rpm]	720
Output [kW]	397
Compression ratio	1:14
Brake mean effective pressure [MPa]	1.47
**Water brake**	
Type	DPx/7D
Power range [kW]	30–2200
Speed [rpm]	300–2100
Dynamometer constant k [Nm/N]	1.104
force sensor FT-5367M range	6.0 kN, accuracy class 0.6%

**Table 2:** Ship main particulars.

	**LOA**	**BOA**	**Draught**	**DWT**
Hull	150.37 m	23.40 m	6.015 m	7330 t
Lanes (LxWxH)	1650 m	3.10 m	4.90 m	1 Ramp

**Table 3:** Main engines specification.

	**Engine Type**	**MCR**	**Engine Speed**	**Bore/Stroke**
2 x	Sulzer 8ZAL 40S	5760 kW	510 RPM	400/560 mm

**Table 4:** Gearbox and power take out (PTO) data.

	**Type**	**Ratio**	**Propeller Speed**	**PTO Power**
2 x	Renk HSU-800	3.083	165.41 RPM	850 kW
